# Application of Smart Packaging in Fruit and Vegetable Preservation: A Review

**DOI:** 10.3390/foods14030447

**Published:** 2025-01-29

**Authors:** Liuzi Du, Xiaowei Huang, Zhihua Li, Zhou Qin, Ning Zhang, Xiaodong Zhai, Jiyong Shi, Junjun Zhang, Tingting Shen, Roujia Zhang, Yansong Wang

**Affiliations:** 1 School of Food and Biological Engineering, School of Agricultural Equipment Engineering, Jiangsu University, 301 Xuefu Rd., Zhenjiang 212013, China; duliuzi@163.com (L.D.); huangxiaowei@ujs.edu.cn (X.H.); 19599960979@163.com (Z.Q.); zhangning980409@163.com (N.Z.); zhai_xiaodong@ujs.edu.cn (X.Z.); shi_jiyong@ujs.edu.cn (J.S.); junjun_5457@ujs.edu.cn (J.Z.); shentingtingstt@ujs.edu.cn (T.S.); 1000005191@ujs.edu.cn (R.Z.); 2 College of Food Science and Engineering, Nanjing University of Finance and Economics, Collaborative Innovation Center for Modern Grain Circulation and Safety, 128 North Railway Street, Gulou District, Nanjing 210023, China; 3 Focusight (Jiangsu) Technology Co., Ltd., No. 258-6 Jinhua Road, Wujin Economic Development Zone, Changzhou 213146, China; rogerswang@focusight.net

**Keywords:** fruit and vegetable preservation, smart packaging, sensor technology, internet of things, degradable materials, food safety, sustainable development

## Abstract

The application of smart packaging technology in fruit and vegetable preservation has shown significant potential with the ongoing advancement of science and technology. Smart packaging leverages advanced sensors, smart materials, and Internet of Things (IoT) technologies to monitor and regulate the storage environment of fruits and vegetables in real time. This approach effectively extends shelf life, enhances food safety, and reduces food waste. The principle behind smart packaging involves real-time monitoring of environmental factors, such as temperature, humidity, and gas concentrations, with precise adjustments based on data analysis to ensure optimal storage conditions for fruits and vegetables. Smart packaging technologies encompass various functions, including antibacterial action, humidity regulation, and gas control. These functions enable the packaging to automatically adjust its internal environment according to the specific requirements of different fruits and vegetables, thereby slowing the growth of bacteria and mold, prolonging freshness, and retaining nutritional content. Despite its advantages, the widespread adoption of smart packaging technology faces several challenges, including high costs, limited material diversity and reliability, lack of standardization, and consumer acceptance. However, as technology matures, costs decrease, and degradable smart packaging materials are developed, smart packaging is expected to play a more prominent role in fruit and vegetable preservation. Future developments are likely to focus on material innovation, deeper integration of IoT and big data, and the promotion of environmentally sustainable packaging solutions, all of which will drive the fruit and vegetable preservation industry toward greater efficiency, intelligence, and sustainability.

## 1. Introduction

In recent years, the global production and consumption of fruits and vegetables have increased rapidly, driven by the growth of the global population and improvements in eating habits and health awareness. This trend is expected to continue in the future. However, the rise in food waste presents serious environmental, economic, and nutritional challenges [1,2]. Fruits and vegetables are highly valued by consumers for their water content, vitamins, minerals, dietary fiber, and other essential nutrients [3,4,5]. The World Health Organization recommends consuming at least 400 g of fruits and vegetables daily to reduce the risk of cardiovascular diseases and certain cancers [6]. However, fresh fruits and vegetables undergo various changes before and after harvest and during storage and transportation due to factors such as water loss, metabolic processes, and microbial infections [7,8,9,10]. These changes affect the external characteristics (e.g., firmness, color, volume) and internal components (e.g., sugars, acidity, phenolic compounds), ultimately leading to spoilage and a loss of flavor and economic value [11,12,13,14]. Therefore, finding effective ways to extend the shelf life of fruits and vegetables and slow down their deterioration has become an urgent challenge for the global agricultural and food industries.

**Figure 1:**
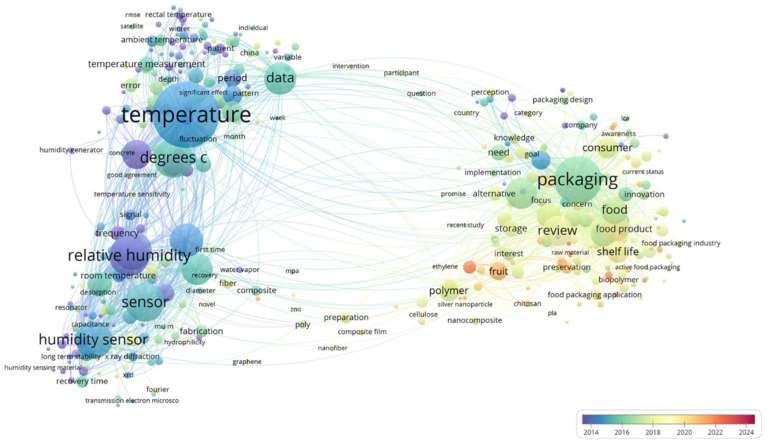
Frequency and co-occurrence of smart packaging/fruit and vegetable freshness-keeping keywords.

To effectively improve the quality of fruit and vegetable products, smart packaging has emerged as a promising solution [15,16,17,18]. With growing global concerns regarding food safety, environmental protection, and sustainable development, the application of smart packaging technology in modern fruit and vegetable preservation has become a key driver of innovation in both agriculture and the food industry [19,20,21,22]. Figure 1 illustrates the time evolution, research directions, and key areas of smart elligent packaging technology in the context of fruit and vegetable preservation over recent years. Early studies (2014–2018) primarily focused on temperature and humidity monitoring technologies, as well as the performance optimization of related sensors. In contrast, current research (2020–2024) has shifted its focus toward the material properties of food packaging and its effects on preservation. This shift is particularly evident in advancements in technology (e.g., enhanced sensitivity requirements for temperature and humidity monitoring), materials (e.g., the use of polymer materials and nanotechnology to improve packaging performance), and applications (e.g., fruit and vegetable preservation) [23,24,25,26]. Smart packaging integrates advanced sensing technology, information technology, and the IoT to dynamically adjust internal conditions based on the state of the food and changes in the external environment [27,28,29]. Compared to traditional packaging, smart packaging enables real-time monitoring and control of environmental variables—such as temperature, humidity, and oxygen concentration—thereby helping to extend the freshness of fruits and vegetables, reduce waste, and improve both product quality and food safety [28]. Additionally, it offers benefits in terms of environmental sustainability and traceability, promoting the modernization and sustainable development of the fruit and vegetable industry. Currently, commonly used smart packaging technologies include smart labels and sensors, breathable films with variable permeability, and materials for temperature and humidity regulation. These technologies not only protect the outer layer of fruits and vegetables but also enhance the storage and transportation processes throughout the entire supply chain [30,31,32,33,34].

The application of active and intelligent food packaging in the preservation of fruits and vegetables is of significant importance [35,36,37]. This is primarily reflected in its ability to extend the shelf life of produce and reduce food waste. While traditional preservation methods such as refrigeration and freezing can effectively prolong storage time, they often compromise the quality of fruits and vegetables [38,39,40]. In contrast, smart packaging dynamically adjusts the internal environment based on real-time monitoring of temperature, humidity, gas concentrations, and other relevant factors, thereby maintaining and even improving the quality of produce [41,42,43,44]. Another key benefit of smart packaging is its contribution to food safety and quality management. By continuously monitoring environmental conditions such as temperature and humidity, smart packaging ensures that fruits and vegetables are stored under optimal conditions. Additionally, it can assess product quality by tracking indicators such as maturity, acidity, and sugar content, thereby reducing the risk of safety issues arising from spoiled or degraded products [45,46,47,48]. Smart packaging also supports traceability and enhances transparency. By embedding QR codes and anti-counterfeiting marks, packaging can provide real-time access to information such as product origin, production dates, and transportation routes. This capability helps prevent the introduction of substandard products into the market and fosters greater consumer trust [49,50]. Furthermore, smart packaging promotes environmental sustainability. Traditional plastic packaging, widely used in food preservation, contributes significantly to environmental pollution due to its non-degradability. Innovations in smart packaging, such as biodegradable materials, plant-based substrates, and recycled components, offer effective alternatives that reduce plastic use and lessen the environmental burden. The integration of smart packaging technology in fruit and vegetable preservation represents an inevitable trend in the evolution of the modern food industry. It plays a crucial role in ensuring food safety, reducing waste, improving consumer experiences, and promoting environmental sustainability [17,25,51]. Through precise monitoring, real-time adjustments, and data traceability, smart packaging provides robust support for the preservation and quality management of fruits and vegetables [52,53]. As technology continues to advance, smart packaging will become increasingly central to the fruit and vegetable industry, driving the global food sector toward more smart, sustainable, and environmentally friendly practices.

This review aims to provide a comprehensive overview of the principles underlying smart packaging technology, with particular emphasis on its practical applications and the challenges associated with implementing these technologies in diverse environments. This review will also explore the critical role of food packaging and highlight recent advancements in packaging technologies and materials. Additionally, it seeks to offer technical guidance for the development of packaging solutions specifically designed for fruit and vegetable preservation, thereby minimizing food waste. The discussion will demonstrate how packaging can play an integral role in enhancing the efficiency and sustainability of the global food supply chain.

## 2. The Technical Principle of Smart Packaging

### 2.1. Smart Packaging Classification

**Figure 2:**
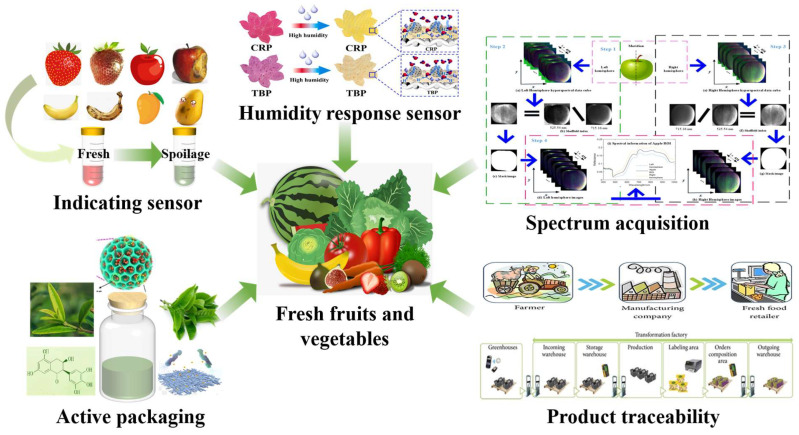
Smart packaging in the classification of fruits and vegetables.

With the continuous advancement of science and technology, smart packaging technology has become increasingly diverse. As shown in Figure 2, based on their working principles, it is mainly divided into two categories: active packaging and intelligent packaging.

Active packaging involves the incorporation of various gas absorbents and release agents within the packaging material, enabling interaction with the product or its surrounding environment to extend shelf life, enhance quality, and maintain freshness. This approach allows for the regulation of factors such as oxygen, carbon dioxide, and humidity within the packaging, creating an optimal environment for the storage of fresh-cut fruits and vegetables. The primary advantage of active packaging lies in its ability to control these environmental factors, inhibiting microbial growth, reducing oxidation reactions, and consequently prolonging the shelf life of food. Active packaging materials typically contain components with adsorption and release capabilities, which can interact with moisture, gases, or other elements within the packaging environment. Common active packaging technologies include oxygen absorbers, carbon dioxide releasers, humidity regulators, and antimicrobial agents. For instance, oxygen absorbers efficiently remove oxygen from the package, thereby preventing oxidative spoilage and delaying the degradation of food; humidity regulators absorb excess moisture, thereby preventing mold and bacterial growth and preserving the freshness of the food; antimicrobial agents effectively inhibit microbial proliferation within the packaging, reducing the risk of contamination and spoilage. In the context of fruit and vegetable preservation, the application of active packaging is particularly critical. By regulating the gas composition and humidity levels within the package, active packaging optimizes the storage conditions of fruits and vegetables, minimizing water evaporation and slowing the deterioration of freshness. For example, certain active packaging materials can lower the respiration rate of fruits and vegetables by absorbing excess carbon dioxide or adjusting the oxygen concentration, thereby delaying the ripening process and maintaining both their taste and nutritional value.

Intelligent packaging refers to the integration of sensors, indicators, or QR codes within the packaging system to monitor and communicate information regarding the condition of products, such as freshness, quality, and traceability. These technologies enable real-time tracking of product status, providing valuable data on factors like temperature, humidity, and the presence of contaminants, thereby enhancing the ability to assess and ensure product integrity throughout its supply chain. Intelligent packaging systems are generally classified into three categories: indicator-based; sensor-based; and data carrier-based packaging. Indicator-based intelligent packaging provides real-time feedback on product status through physical or chemical changes. It is commonly used in cold chain logistics and for perishable foods, where it helps monitor environmental variables such as temperature and humidity during transportation, thereby preventing quality degradation. Sensor-based intelligent packaging integrates sensors capable of monitoring multiple environmental parameters, such as temperature, humidity, and gas concentration. These systems offer timely feedback on storage and transportation conditions, thereby enhancing product quality control and supply chain management efficiency. Additionally, carrier-based intelligent packaging data store and transmit product information, including production dates and shipping records, through embedded QR codes, Radio Frequency Identification (RFID), or Near Field Communication (NFC) tags. This enhances product transparency, traceability, and consumer trust while optimizing inventory management [54,55,56,57]. In conclusion, although each of these three types of intelligent packaging has distinct technical characteristics and application scenarios, their integration will increasingly contribute to improved product safety, optimized supply chain management, and enhanced consumer experiences.

#### 2.1.1. Active Packaging

Active packaging is an advanced packaging technology that actively interacts with the contents of the package or the external environment. In contrast to traditional passive packaging, active packaging not only provides protection but also regulates the internal atmosphere of the package (e.g., oxygen, humidity, carbon dioxide) or interacts with food through specific substances or technologies, thereby extending the freshness, maintaining the quality, and potentially enhancing the safety of the product. For instance, to address the challenges of spoilage and browning in fresh-cut vegetables, Zhang et al. [58] developed a cost-effective and environmentally friendly active packaging by incorporating carbon quantum dots (CQDs) extracted from lemons into guar gum (GG) and sodium alginate (SA) films. This active packaging demonstrated strong antioxidant and antibacterial properties, effectively inhibiting browning in fresh-cut vegetables. Similarly, to extend the freshness of strawberries, Bian et al. [59] successfully developed a nano-cellulose-based fruit cling film by combining lignin and polyphenols. This active packaging exhibited notable antioxidant, antibacterial, and UV-shielding properties, which significantly prolonged the shelf life of strawberries. Overall, active packaging technology optimizes the storage conditions of food by regulating the internal environment of the package, thereby reducing food waste and extending shelf life. It represents a promising and innovative advancement in the field of food packaging.

#### 2.1.2. Indicative Intelligent Packaging

Indicating intelligent packaging refers to a new type of packaging that integrates sensing technology and visual indicators, building upon traditional packaging. It primarily functions by responding to characteristic conditions of the packaging environment (such as temperature, humidity, pH, oxygen concentration, etc.) and target substances (including volatile nitrogen compounds, carbon dioxide, aldehydes, etc.). This enables the packaging to monitor food quality during storage and sales. Depending on the type of detection, indicating intelligent packaging can be categorized into three types: environmental indicator packaging, which monitors the impact of the external environment on the product; quality indicator packaging, which tracks real-time changes in product quality; and time indicator packaging, which assesses temperature changes during storage or transportation using technologies such as time–temperature integration sensors (TTI). This helps determine whether the product has exceeded the safe usage range.

During the storage, transportation, and sale of fresh food, characteristic volatile substances are generated as the freshness quality deteriorates. These changes can be monitored to assess the quality of fresh fruits and vegetables. The internal signal–source response indicator packaging is extensively employed in the research of freshness indicators for fresh fruits and vegetables. Zhang et al. [60] loaded phenol red and thymol blue onto Co-MOF materials to prepare an indicator packaging system. During the storage of fresh-cut apples, the color of the indicator film changed from yellow to blue as the freshness of the apples decreased. Zhan et al. [61] developed a pH-sensitive freshness label based on red cabbage extract, where the color of the mushroom membrane shifted from red-brown to light brown as the freshness of the mushrooms declined during storage. Maftoonazad et al. [62] created an electrospun pH biosensor enriched with purple cabbage anthocyanins, which exhibited a color change from blue to purple as the freshness of fresh jujubes decreased during storage. Zhang et al. [63] prepared a bifunctional polyvinyl alcohol/polyvinylimine/methylene blue nanofiber indicator film. During the storage of fresh-cut fruits (strawberries, apples, watermelon, and papaya), the color of the indicator film transitioned from blue to white as the freshness of the fruits declined. Chen et al. [64] mixed methyl red and bromothymol blue solutions (in a 3:2 ratio) to prepare an indicator film. During the storage of freshly cut green peppers, the color of the indicator film changed from yellowish–green to orange as the freshness of the peppers decreased.

#### 2.1.3. Sensing Intelligent Packaging

Sensing intelligent packaging utilizes electronic devices to detect changes in the physical or chemical properties (such as color, conductivity, luminescence, etc.) of packaging materials during food quality monitoring and converts these changes into electrical signals, thereby enabling the identification of food quality. Based on different working principles, sensing intelligent packaging can be classified into chemical sensor-based and biological sensor-based systems. Chemical sensor-based intelligent packaging involves embedding a response material onto the packaging material; when the concentration of a target substance changes, detectable alterations in the physical properties of the packaging material (e.g., electrical conductivity, color) occur, reflecting the quality of the food. Although sensing intelligent packaging demonstrates significant potential in the field of food quality monitoring, with high specificity, low detection limits, and excellent reproducibility, several challenges remain. First, the sensor material’s response component may migrate to the food surface during monitoring, posing potential food safety risks. Second, the high development and application costs of sensors limit their widespread use in mass production and commercialization.

Wang et al. [65] developed fluorescent sensor arrays using curcumin, puerarin, and fisetin. By exploiting the different volatile substances produced by various fruits and vegetables during the deterioration process (e.g., spinach produces alkaline volatile compounds, while sweet corn produces acidic ones), they combined the fluorescence sensor array with a deep convolutional neural network (DCNN) to detect the acidity or alkalinity of volatile organic compounds. This approach enabled non-destructive, real-time, and accurate classification of freshness. Mahata et al. [66] synthesized SnO_2_ nanosensors via a low-temperature hydrothermal method to effectively monitor fruit freshness by analyzing the volatile substances emitted by apples, pomegranates, grapes, and oranges during storage. Fatchurrahman et al. [67] employed fluorescence spectroscopy, along with measurements of hardness, color, and other indicators, to assess the freshness of green peppers during storage. They also combined hyperspectral imaging to analyze the surface structure of the peppers, providing a comprehensive method for freshness monitoring.

#### 2.1.4. Data Carrier-Type Intelligent Packaging

Data carrier intelligent packaging refers to packaging technology that enables the monitoring, traceability, and information management of food, pharmaceuticals, and other products by embedding or attaching intelligent components capable of storing and transmitting data within the packaging materials. This type of intelligent packaging typically utilizes technologies such as electronic labels, RFID (radio frequency identification), two-dimensional barcodes, and NFC (near-field communication) to store and transmit information. These technologies allow for the recording and updating key information throughout the production, transportation, and storage processes. Consumers, supply chain managers, and other stakeholders can access this information via smart devices (e.g., smartphones, scanners) to enable real-time monitoring of products, quality traceability, expiration date tracking, and other functions. For example, Tao et al. [68] developed a multi-information fusion RFID sensor using nano-sensitive materials. The sensor’s detection sensitivity for temperature, carbon dioxide, and ethanol concentration was found to be 0.25 dB/°C, 0.011 dB/ppm, and 0.65 MHz/ppm, respectively. Additionally, Ben Hattab et al. [69] designed an RFID system to study the ripening, bruising, and freshness of mangoes by analyzing the dielectric properties of Tommy Atkins Mangoes.

### 2.2. Smart Packaging Materials

Smart packaging materials incorporate advanced technologies and material science to enable packaging to possess functions such as perception, response, and information transmission. These capabilities enhance product safety, freshness, convenience, and the overall consumer experience. Common smart packaging materials are shown in Table 1.

#### 2.2.1. Temperature and Humidity-Sensitive Materials

Thermal labels are designed to change color in response to temperature variations. For instance, temperature-sensitive packaging can change color when it exceeds a specified temperature threshold, providing alerts to consumers or supply chain managers about whether the temperature falls outside the appropriate range. This feature is especially useful for products with stringent temperature control requirements, such as food and pharmaceuticals. Similarly, humidity indicator cards and sensors are capable of detecting changes in humidity levels inside or outside the package. Certain humidity-sensitive materials, such as humidity indicator cards, undergo color changes in response to variations in ambient humidity, helping to assess whether the product’s storage conditions are appropriate. Zhang et al. [71] developed a humidity-sensitive, color-changing polyester fabric induced by the pH indicators cresol red and thymol blue via screen printing. As humidity increased, the film exhibited a gradual color change from red to yellow. Karzarjeddi et al. [70] incorporated cellulose nanofibers and ionic liquids into a highly porous hydrothermochromic foam to create a flexible, temperature-responsive fiber film. As the temperature rose, the film gradually transitioned from light green to blue.

#### 2.2.2. Electronic Labels and Information Transfer Materials

To effectively provide real-time feedback on food information, various informational labels have been extensively developed and applied. Common RFID tags are primarily used for automatic product identification, real-time tracking, and preventing the loss or theft of goods. QR codes and NFC labels enable consumers to scan the label on a package with their smartphones to access product details, such as production date, ingredients, and usage instructions. Additionally, electronic shelf labels (ESL) can automatically update pricing, promotions, and other information via wireless transmission, eliminating the need for manual label replacement. Alfi et al. [80] developed a light-emitting polyvinyl alcohol/carboxymethyl cellulose (PVA/CMC) nanocomposite material enriched with lanthanides that provides photochromic, ultraviolet protection, antibacterial activity, and hydrophobic properties. This material also enables anti-counterfeiting features through photochromic changes. Wang et al. [81] demonstrated that the integration of two-dimensional codes and electronic tags can effectively automate and digitalize the management of kiwifruit cultivation, enabling full-process traceability and significantly enhancing the efficiency of kiwifruit breeding management.

#### 2.2.3. Self-Healing Materials

During storage and transportation, fruits and vegetables are susceptible to microbial invasion due to packaging damage and other factors, leading to food spoilage. Therefore, materials with self-healing properties are crucial. Self-healing coatings and films are designed to automatically repair cracks or breaks in the packaging surface, while self-healing plastics can autonomously mend minor cracks or damage after they occur. Du et al. [82] developed a self-healing edible coating composed of chitosan (CS) and sodium alginate (SA), which maintains its barrier properties and mitigates the loss of freshness caused by coating damage, thereby effectively extending the shelf life of strawberries. Wang et al. [83] introduced a self-healing hydrogel packaging film based on nanocellulose (CNF), polyvinyl alcohol (PVA), and ZIF-8 embedded with curcumin (Cur@ZIF-8). This film exhibits excellent water vapor barrier properties, mechanical strength, antibacterial activity, antioxidant effects, and ammonia sensitivity, and it can effectively extend the shelf life of fish by up to 9 days.

#### 2.2.4. Antibacterial Freshness-Keeping Materials

Active packaging materials inhibit the growth of bacteria and microorganisms by incorporating antibacterial agents, such as silver ions, copper ions, and nanomaterials, into the packaging surface. This approach can extend the shelf life of food, pharmaceuticals, and other products, particularly in food packaging. Additionally, the inclusion of antioxidant ingredients within certain packaging materials can effectively prevent oxygen from penetrating the package and reacting with the contents, thereby preserving food freshness. Huang et al. [84] developed an antibacterial indicator bifocal fiber membrane composed of zein/gelatin/carvacrol and PVA/chitosan/anthocyanin via electrospinning technology. This membrane not only extends the shelf life of fish but also monitors its freshness by undergoing a color change from red to blue. Wang et al. [85] prepared a freshness-keeping film by incorporating essential oils into chitosan and applied it to loquat fruit. This film effectively delayed weight loss, retained high levels of total soluble solids and ascorbic acid, and reduced the rot index and respiratory rate.

#### 2.2.5. Biodegradable Materials

With the growing emphasis on environmental protection, many active packaging materials have shifted toward using biodegradable substances, such as polylactic acid (PLA) and polyhydroxyfatty acid ester (PHA). These materials naturally degrade after use, helping to reduce plastic pollution. Similarly, some active packaging solutions incorporate plant-based extracts (e.g., cornstarch, sugarcane, wood pulp) as raw materials, which not only promote sustainability but also decrease reliance on traditional petroleum-based plastics. Riahi et al. [86] synthesized chitosan-based carbon quantum dots with multifunctional properties through a hydrothermal method and incorporated them into carboxymethyl cellulose to prepare a functional film. This film exhibited excellent oxidation resistance and strong antibacterial activity and was applied to the surface of lemons. He et al. [87] prepared an antibacterial carboxymethyl cellulose (CMC) nanocomposite film containing montmorillonite (MMT) and ε-poly(L-lysine) (ε-PL) via a solution-casting method, using it as a preservation coating for strawberries, which extended their shelf life by 2 days.

#### 2.2.6. Nanotechnology Packaging Materials

Nanomaterials are widely utilized in packaging to enhance protective properties and extend shelf life. For instance, silver nanoparticle coatings exhibit strong antibacterial properties, effectively inhibiting the growth of microorganisms. Additionally, nanofiber films provide superior gas barrier performance in food packaging, preventing the ingress of oxygen, moisture, and other factors that could compromise food freshness. Wang et al. [88] developed a biopolymer film composed of chitosan, potato protein, linseed oil, and ZnO nanoparticles for raw meat storage. Experimental results demonstrated that this film significantly extended shelf life, maintained excellent sensory properties during 7 days of storage, and effectively reduced the rate of pH increase and bacterial growth. Song et al. [89] created two types of hydrophobic electrospun fluorescence sensors using polyvinylidene fluoride (PVDF) as the film-forming polymer and double-emission carbon quantum dots (CQDs) as fluorescent probes for real-time monitoring of food deterioration. When applied to beef, pork, and shrimp, the sensors exhibited visible fluorescence color changes from yellowish–green to blue, indicating food spoilage.

## 3. Application of Smart Packaging in Fruit and Vegetable Preservation

With the continuous improvement in consumer demands for food quality, particularly in the preservation and safety of fruits and vegetables, smart packaging technology has gradually become a critical means of enhancing the shelf life of these foods. Active packaging not only extends the shelf life of fruits and vegetables but also improves food safety, reduces waste, and addresses modern society’s growing needs for sustainability and environmental protection. Key application areas of smart packaging technology in fruit and vegetable preservation include air-regulated packaging, temperature, and humidity-controlled packaging, antibacterial and freshness-preserving packaging [90,91,92], and biodegradable packaging, as summarized in Table 2. 

### 3.1. Temperature and Humidity Intelligent Packaging

The application of Temperature and Humidity Intelligent Packaging (T&H intelligent packaging) in fruits and vegetables involves the intelligent monitoring and adjustment of the temperature and humidity environment within the packaging to ensure the optimal freshness and quality of produce during transportation and storage. This technology integrates modern sensors, smart materials, and control systems to continuously monitor temperature and humidity changes inside the packaging, automatically adjusting them in real time to maximize the shelf life of fruits and vegetables and reduce waste. For most fruits and vegetables, maintaining proper humidity is crucial to preserving freshness. Intelligent packaging can regulate humidity levels to prevent excessive water loss or absorption, thereby reducing issues such as shriveling, rotting, and other forms of deterioration. Wu et al. [93] developed a hydrophobic ethyl cellulose film enriched with 1-methylcyclopropene (1-MCP), which controlled the release rate of 1-MCP by adjusting the humidity within the package. This approach effectively reduced the ethylene concentration, delayed the softening, browning, and weight loss of mushrooms, and preserved the integrity of their cell membranes. It also maintained the levels of ascorbic acid and soluble solids while inhibiting the activity of enzymes involved in ethylene synthesis. Moggia et al. [100] created an Accelerated Dehydration (DAD) suspension device to evaluate its ability to detect variations in fruit dehydration. Blueberries harvested at early, peak, and late stages were exposed to three controlled relative humidity levels (30%, 65%, and 96%). The results demonstrated that both the fruit’s stage of harvest and the humidity treatment, as well as their interaction, significantly affected the weight loss of the fruit at each stage. The application of temperature and humidity intelligent packaging technology offers a more efficient preservation solution for the fruit and vegetable industry, extending shelf life, reducing food waste, and better meeting consumer demand for fresh, high-quality produce.

### 3.2. Antibacterial Freshness-Keeping Active Packaging

The application of antibacterial freshness-keeping packaging for fruits and vegetables is a technology that delays spoilage, extends shelf life, and maintains freshness and quality through the use of packaging materials or technologies with antibacterial properties. These materials inhibit or kill the growth of bacteria, molds, and other microorganisms, reducing microbial contamination that can lead to deterioration and quality loss during storage and transportation. Antibacterial packaging can slow the growth of microorganisms, thereby reducing the rate of spoilage and extending shelf life. This technology is particularly effective for highly perishable fruits and vegetables, such as strawberries, grapes, and cherries. Zhao et al. [94] developed a biodegradable, antibacterial composite film by incorporating the porphyrin metal–organic framework MOF-545 into polycaprolactone (PCL). This film serves as a highly efficient photosensitizer under LED light irradiation, effectively killing 96% of microorganisms when applied to fresh-cut apples. This application significantly delays apple discoloration and water loss. Tavassoli et al. [95] fabricated a smart film by loading silver metal–organic framework (Ag-MOF) nanoparticles with lactoferrin (LAC) into a matrix of methyl cellulose and chitosan nanofibers. Due to its strong antibacterial and antioxidant properties, this film maintains the fresh appearance of apples for at least 7 days when applied. In summary, antibacterial freshness-keeping packaging technology provides an effective solution for the fruit and vegetable industry, not only extending shelf life and reducing microbial contamination but also minimizing food waste and enhancing the consumer purchasing experience. With advances in technology and material innovation, the future of antibacterial freshness-keeping packaging is expected to see broader adoption in the fruit and vegetable sector, further advancing the development of food preservation technologies.

### 3.3. Biodegradable Active Packaging

Biodegradable packaging refers to packaging materials, either natural or synthetic, that can be broken down by microorganisms, humidity, temperature, and other environmental factors, eventually converting them into harmless substances. The application of biodegradable packaging in the fruit and vegetable sector primarily addresses the environmental pollution caused by traditional plastic packaging while enhancing the preservation of produce and reducing food waste. Traditional plastic packaging can take centuries to degrade, during which harmful substances may be released. In contrast, biodegradable packaging decomposes in a relatively short period, thereby alleviating environmental burden and aligning with sustainable development principles. Moreover, many biodegradable materials exhibit excellent airtightness and moisture control properties, effectively extending the shelf life of fruits and vegetables. For instance, certain composite materials can block oxygen, thereby slowing the oxidation process and prolonging freshness. Nie et al. [101] extracted a protein mixture containing polyphenols, flavonoids, and tea saponins from oil tea residue, combining it with chitosan to produce biodegradable films that significantly extended the shelf life of perishable fruits such as strawberries. Regmi et al. [102] extracted cellulose from soybean residues and crosslinked it with calcium ions and glycerin to create biodegradable films, which effectively prolonged the shelf life of raspberries to the sixth day. This suggests that soybean shell cellulose films are advantageous in minimizing post-harvest losses and addressing plastic-related environmental issues, supporting the principles of a circular bioeconomy. In conclusion, the use of biodegradable packaging in fruits and vegetables presents an environmentally friendly solution that extends produce freshness, reduces food waste, and mitigates the negative impacts of plastic pollution. With ongoing technological advancements and growing consumer awareness of environmental protection, biodegradable packaging is poised for broader adoption, potentially transforming the fruit and vegetable packaging industry.

### 3.4. Application of Nanotechnology in Fruit and Vegetable Preservation

The application of nanotechnology in the preservation of fruits and vegetables has emerged as a pivotal area of research within the food industry in recent years. One promising approach is the use of nano-coating technology, which involves applying a nanometer-thin coating or film to the surface of fruits and vegetables. This protective layer exhibits high permeability to gases while also providing resistance to oxygen, thereby reducing the exchange of oxygen and water, slowing the respiration rate of produce, and delaying the onset of decay. Furthermore, the nano-coating acts as a barrier against pathogenic microorganisms, inhibiting the growth of bacteria and fungi and thereby enhancing the preservation of fruits and vegetables. In addition to coating technologies, nanoparticles are increasingly utilized as antimicrobial agents. Materials such as nanosilver and nano-titanium dioxide, known for their potent antibacterial properties, can release bactericidal ions upon contact with fruit and vegetable surfaces, thereby suppressing microbial growth. These nano-antibacterial agents not only demonstrate high efficacy but also reduce reliance on traditional chemical preservatives, offering a safer and more environmentally sustainable alternative. For example, Ding et al. [97] integrated polytannic acid (PTA) with TiO_2_ nanoparticles to develop a multifunctional chitosan film with visible light-responsive photocatalytic properties. When applied to kiwifruit and grapes, this film reduced respiration and transpiration rates in kiwifruit, slowed browning, and lowered ethylene levels in the storage environment, thereby extending shelf life. Similarly, Li et al. [98] developed a multifunctional food packaging system based on ε-poly-L-lysine (ε-PL) and rhamnolipid (RL), created through electrostatic self-assembly. This packaging, when applied to various fruits, successfully extended the storage life of strawberries by three days. Jiang et al. [99] synthesized a ZIF-8/quaternized chitosan (CS) nanocomposite membrane with a “nanobarrier” structure through a one-pot co-crystallization process. Compared with control groups, this membrane significantly extended the shelf life of fruit by up to eight days. In conclusion, the application of nanotechnology in the preservation of fruits and vegetables not only effectively extends their shelf life but also improves their safety and nutritional value. With ongoing advancements in nanotechnology, its role in food preservation is expected to grow, offering consumers fresher, safer food options.

## 4. Advantages and Challenges of Smart Packaging in Fruit and Vegetable Preservation

### 4.1. Advantages of Smart Packaging in Fruit and Vegetable Preservation

**Figure 3:**
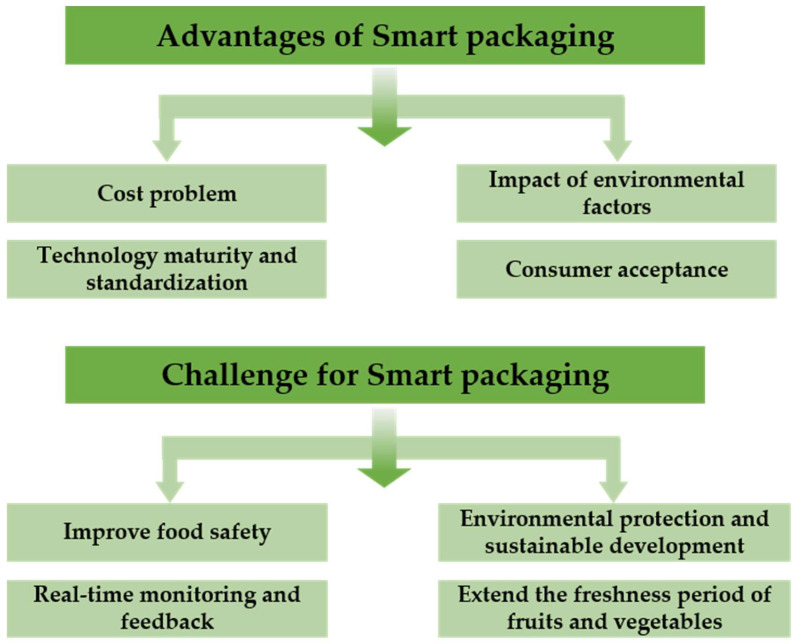
Advantages and challenges of smart packaging in fruit and vegetable preservation.

As perishable foods, fruits and vegetables are highly susceptible to various factors such as temperature, humidity, and gas composition, which can lead to significant changes in their quality. Traditional preservation methods, including refrigeration, modified atmosphere packaging, and chemical preservatives, are effective in extending the shelf life of fruits and vegetables but still have limitations. In recent years, the rapid development of smart packaging technology has provided new solutions for the preservation of fruits and vegetables. Smart packaging allows for real-time monitoring and regulation of the storage environment by integrating sensors, smart membranes, gas control systems, and other technologies. This approach effectively extends the freshness of fruits and vegetables, reduces waste, and enhances supply chain efficiency. Details are shown in Figure 3.

#### 4.1.1. Extend the Freshness Period of Fruits and Vegetables

One of the most significant advantages of smart packaging technology is its ability to extend the shelf life of fruits and vegetables by real-time monitoring and regulation of environmental factors. For instance, smart packaging systems, integrated with temperature and humidity sensors, can continuously monitor the storage conditions of fruits and vegetables during transportation and storage, ensuring that they remain in optimal preservation environments, thereby effectively extending their shelf life [103]. Additionally, the incorporation of active substances and biodegradable materials can significantly delay microbial contamination during the storage process, further prolonging the freshness and shelf life of fruits and vegetables [104].

#### 4.1.2. Real-Time Monitoring and Feedback

Smart packaging can be equipped with various sensors, such as temperature, humidity, and gas sensors, to enable real-time monitoring of environmental changes both inside and outside the packaging. Through a feedback mechanism, the system can promptly adjust the packaging conditions or activate an alarm. This real-time monitoring not only provides accurate data for the logistics supply chain but also offers consumers critical information, such as whether the fruit is in optimal eating condition [105]. Furthermore, smart packaging can be integrated with cloud platforms via the IoT system, enabling smart management and traceability for producers and distributors of fruits and vegetables [106].

#### 4.1.3. Improve Food Safety

Smart packaging technology can significantly reduce the risk of contamination in fruits and vegetables during transportation and storage. Certain smart packaging materials possess antibacterial, antioxidant, and other functional properties that effectively inhibit the growth of bacteria and mold, thereby reducing the risk of food spoilage. Additionally, the incorporation of gas regulation systems and self-healing materials within the packaging can protect fruits and vegetables from external environmental damage, ensuring the safety and integrity of the product [107,108,109].

#### 4.1.4. Environmental Protection and Sustainable Development

With the growing emphasis on environmental sustainability, bio-based films have gained significant attention in food packaging due to their biodegradable properties. Many smart packaging materials are derived from degradable substrates, such as polylactic acid (PLA), polysaccharides, proteins, and lipids, which are used to create biodegradable smart films with diverse functionalities, including preservation, freshness monitoring, and antioxidant effects. These materials not only facilitate the effective monitoring of freshness and smart preservation of fruits and vegetables but also contribute to the reduction in environmental pollution [110,111,112]. This characteristic is particularly crucial in the field of fruit and vegetable preservation, as packaging is often discarded after consumption. The use of degradable materials can, thus, help mitigate plastic waste.

Smart packaging technologies have significantly impacted the food industry, consumer behavior, and human health. These innovations facilitate real-time monitoring of environmental factors such as temperature, humidity, and gas composition, thereby preserving the freshness and quality of food. This extends the shelf life of products and reduces food waste. Furthermore, when integrated with the Internet of Things (IoT), smart packaging enables real-time product tracking and data analysis, ensuring comprehensive control over the supply chain—from production to retail—and maintaining food safety and quality. In terms of consumer behavior, smart packaging enhances confidence in food quality by providing critical real-time information, such as expiration dates and storage conditions. This transparency influences purchasing decisions. Additionally, smart packaging offers personalized services, such as health tips, nutritional information, and recipe suggestions, allowing consumers to make more informed, health-conscious choices. From a public health perspective, smart packaging has the potential to reduce foodborne illnesses and other health risks by continuously monitoring food status and ensuring its safety. However, certain materials used in smart packaging, particularly those incorporating nanotechnology, may pose health risks if improperly used or over time, with hazardous substances potentially migrating into food. Rigorous safety assessments and regulations are necessary to mitigate these risks. In summary, while smart packaging technologies drive innovation in the food industry, enhance consumer experiences, and promote sustainability, they also present both positive and negative implications for health. Striking a balance between technological advancement and safety is essential to maximize the long-term benefits of these technologies.

### 4.2. Challenges of Smart Packaging in Fruit and Vegetable Preservation

#### 4.2.1. Cost Problem

Although smart packaging technology offers significant advantages in fruit and vegetable preservation, its cost remains higher than that of traditional packaging. Materials used in smart packaging, such as air-regulated films, temperature and humidity sensors, and RFID tags, are associated with high manufacturing costs, which can impose a substantial economic burden on small and medium-sized manufacturers and low-cost markets. Therefore, reducing the production and application costs of smart packaging remains a key challenge to the widespread adoption of this technology.

#### 4.2.2. Technology Maturity and Standardization

The emergence of advanced smart packaging materials has significantly enhanced food safety and consumer convenience. However, the application of these materials, particularly nanomaterials, necessitates careful consideration of potential safety and toxicity concerns. Nanomaterials, for instance, can enter the human body via dermal contact, inhalation, or ingestion, posing potential health risks. Research has indicated that certain nanomaterials, such as nanosilver and titanium dioxide, may induce oxidative stress, cause cellular damage, and have detrimental effects on the immune system and internal organs. Furthermore, nanomaterials incorporated into food packaging may migrate into food, especially under conditions of high temperature, acidic or alkaline environments, or high fat content, thereby increasing the risk to consumer health. Smart packaging materials typically contain functional substances designed to respond to environmental changes, such as temperature, humidity, or gas concentration. These materials may include chemical additives or active agents that, under specific conditions, can react with or migrate into food, potentially compromising food quality and safety. For example, indicators or sensors embedded in some smart packaging systems may release toxic substances, particularly when the packaging deteriorates or becomes damaged over time. Currently, there is a lack of standardized international frameworks and regulations for the safety assessment of food packaging materials, especially those involving nanomaterials. Food safety standards vary across regions, with some countries imposing stringent restrictions on nanomaterial use, while others have yet to establish clear guidelines. To ensure the safety of these innovative packaging solutions, it is essential to strengthen the development of regulatory frameworks and conduct further scientific research to assess and regulate the use of nanomaterials and smart packaging in food applications. In conclusion, the diverse types and storage requirements of fruits and vegetables highlight the need for increased adaptability and flexibility within smart packaging technologies. This remains a critical issue to address in the advancement of safe and effective smart packaging solutions.

#### 4.2.3. Consumer Acceptance

Despite the technological advantages of smart packaging, consumer acceptance remains a critical factor. Some consumers may harbor concerns about the safety, environmental impact, and other aspects of the new materials and technologies used in smart packaging. Therefore, improving consumer understanding and acceptance of smart packaging through education and public outreach and promoting its widespread adoption represents a significant challenge in the advancement of smart packaging technology.

#### 4.2.4. Impact of Environmental Factors

The effectiveness of smart packaging in fruit and vegetable preservation largely depends on external environmental factors, such as temperature, humidity, and light. However, these factors are often difficult to fully control in practical applications, particularly during long-term transportation and storage. The variability of the external environment can, therefore, impact the performance and efficacy of smart packaging. Consequently, optimizing smart packaging technology to adapt to the complex and dynamic logistics environment is a key focus for future technological development.

## 5. Future Development Direction

**Figure 4:**
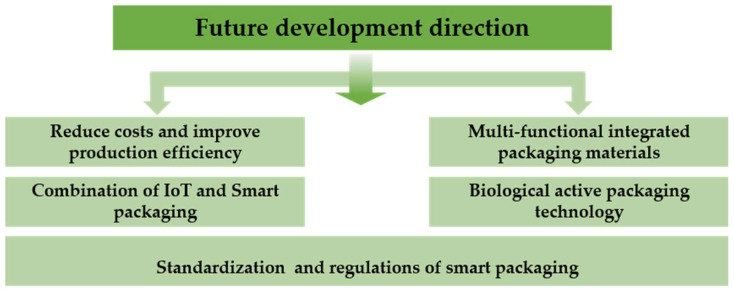
Future development direction.

As consumer demand for the quality and safety of fruits and vegetables continues to rise, smart packaging, as an innovative preservation technology, is emerging as a key solution in the field of fruit and vegetable preservation. Smart packaging not only extends the freshness of fruits and vegetables, preserving their taste and nutritional value, but also enhances transportation efficiency and reduces food waste. Future developments in smart packaging for fruit and vegetable preservation will explore innovations in several areas, including cost-efficiency, multifunctional integrated packaging, the IoT and smart packaging, bio-based smart packaging technologies, and the establishment of smart packaging standards and regulations. As illustrated in Figure 4, with ongoing technological advancements and shifting market demands, smart packaging is poised to not only extend shelf life and improve transportation efficiency but also address environmental and sustainability concerns. In the future, smart packaging will play an increasingly vital role in the preservation of fruits and vegetables, becoming an indispensable component of the food industry.

### 5.1. Reduce Costs and Improve Production Efficiency

Although smart packaging technology offers significant preservation benefits, its high cost remains a major barrier to widespread adoption. Currently, the reliance on advanced materials, sensor technologies, and smart control systems contributes to the elevated costs of smart packaging. However, as smart packaging technology continues to evolve, innovations in materials and manufacturing processes are expected to gradually reduce costs, thereby enhancing its market competitiveness. Concurrently, improvements in the efficiency of smart packaging will help minimize losses of fruits and vegetables during transportation and storage. Through large-scale production and optimized packaging designs, smart packaging can not only ensure long-term preservation but also strike a balance between cost and efficiency, facilitating its adoption in lower-end markets.

### 5.2. Multifunctional Integrated Packaging Materials

As consumer demand for functional foods increases, smart packaging is expected to evolve toward multifunctional integration. Future smart packaging will not only extend the shelf life of fruits and vegetables but also incorporate additional functionalities, such as antibacterial properties, moisture retention, gas regulation, and temperature and humidity monitoring. This integrated smart packaging will automatically adjust the atmosphere, temperature, and humidity inside the package based on the specific needs of different fruits and vegetables, inhibiting the growth of bacteria and mold while also providing real-time monitoring of storage conditions. Innovative materials and technologies, including smart sensors and nanotechnology, may be incorporated into packaging to further enhance its capabilities. For instance, antibacterial packaging based on nanotechnology can effectively prevent surface contamination of fruits and vegetables, while smart packaging combined with temperature and humidity sensors can ensure that fruits and vegetables remain in optimal freshness under varying environmental conditions.

### 5.3. Combination of IoT and Smart Packaging

The integration of IoT technology is expected to be a key trend in the future development of smart packaging. By combining smart packaging with IoT, the entire transportation process of fruits and vegetables can be monitored in real time. IoT technology allows sensors within the packaging to connect to endpoint devices, such as cloud platforms and smartphones, enabling the continuous collection of data on temperature, humidity, and gas composition inside the package. These data can then be used to automatically adjust the packaging environment. For instance, if the sensor detects that the temperature inside the package is too high or the humidity is too low, the system can activate a smart control mechanism to modify the environmental conditions and ensure optimal preservation of the produce. Furthermore, IoT technology enables traceability throughout the entire supply chain, from production to transportation to retail. Consumers can scan the QR code or RFID tag on the package to access information about the product’s origin, transportation route, and storage conditions, thereby increasing consumer confidence in food safety.

### 5.4. Biological Smart Packaging Technology

Biointeligent packaging technology, particularly the use of biodegradable materials, is emerging as a key direction for the future development of smart packaging. With growing environmental awareness, traditional plastic packaging is being increasingly restricted, and biodegradable smart packaging materials offer the potential to reduce environmental pollution without compromising packaging functionality. In the future, biointeligent packaging will not only fulfill the basic functions of conventional packaging but may also integrate biotechnology, smart sensors, and antibacterial features, positioning it as a sustainable solution for fruit and vegetable preservation. For example, by incorporating natural materials such as seaweed extracts and plant fibers, biointeligent packaging can effectively extend the freshness of fruits and vegetables while naturally degrading after use, thus alleviating environmental burdens. Additionally, certain biointeligent packaging systems may further enhance the preservation of produce by releasing specific antibacterial agents or regulating the respiratory rate of fruits and vegetables.

### 5.5. Standardization and Regulations of Smart Packaging

The rapid advancement of smart packaging technology has also highlighted the need for relevant standards and regulations. To ensure the safety and efficacy of smart packaging in fruit and vegetable preservation, it is crucial to develop unified technical standards and regulatory frameworks at an international level. Currently, the standards for smart packaging vary across different countries and regions, leading to technical barriers between markets. In the future, it is possible to establish a globally harmonized smart packaging standard that encompasses various aspects such as materials, functionality, safety, and environmental impact. Furthermore, the enhancement of relevant regulations can facilitate the healthy development of smart packaging technologies, ensuring that their application in the food industry does not pose potential safety risks. Within the framework of these standards and regulations, the widespread adoption of smart packaging will be smoother, fostering greater consumer trust in this emerging technology.

## 6. Conclusions and Prospect

Smart packaging technology, as a significant innovation in the field of fruit and vegetable preservation, holds substantial potential and development prospects. This study provides a comprehensive overview of the current principles and technologies underlying smart packaging, with a particular focus on its materials and applications in fruit and vegetable preservation. It highlights the advantages and challenges associated with the use of smart packaging in this context and explores future research directions and potential developments. The primary goal of smart packaging in the preservation of fruits and vegetables is to extend their shelf life through advanced technological interventions, minimize waste, and enhance the consumer experience. By integrating sensors, smart labels, wireless communication, and other cutting-edge technologies, smart packaging can continuously monitor and record environmental factors such as temperature, humidity, and gas composition. This enables dynamic adjustments to the internal conditions of the packaging, including the regulation of gas composition, release of antibacterial agents, and provision of real-time temperature and humidity data. These smart capabilities not only significantly retard the spoilage process but also enhance traceability and transparency across logistics and sales, ensuring the delivery of fresh and safe products to consumers. However, challenges remain, including the high cost of technology, the diversity and reliability of smart materials, technical standards and regulations, and consumer acceptance. In the future, as the technology matures and evolves, smart packaging is expected to have reduced costs and improved economic feasibility through large-scale production, process optimization, and material innovation. Advances in materials science, particularly the development of biodegradable smart packaging materials, are likely to drive the growth of smart packaging in an environmentally sustainable direction. For instance, the integration of emerging technologies such as plant-based materials, nanotechnology, and adaptive sensors could enhance the interaction between packaging and produce, thereby improving the functionality and adaptability of the packaging. In terms of multifunctional integration, smart packaging will not be limited to preservation but will also include functions such as antibacterial properties, humidity regulation, and gas control. This integrated packaging system can automatically adjust environmental conditions within the packaging based on the specific needs of different fruits and vegetables, effectively reducing the growth of bacteria and mold, extending freshness, and preserving nutrients. Additionally, with the application of the IoT and big data technologies, future smart packaging systems will enable real-time monitoring throughout the entire process, making the storage and transportation of fruits and vegetables traceable. This will enhance food safety, increase consumer trust, and assist manufacturers and retailers in optimizing inventory management and supply chain efficiency.

In summary, the application of smart packaging technology in fruit and vegetable preservation holds significant promise and potential. As technological innovations continue to advance, costs decrease, and materials become optimized, smart packaging is expected to move beyond being a luxury for high-end markets and become a widely accessible tool for improving food quality and reducing waste on a global scale. Looking ahead, smart packaging will increasingly integrate with cutting-edge technologies such as environmental sustainability, the IoT, and data analytics, driving the fruit and vegetable preservation industry toward greater efficiency, sustainability, and intelligence. This integration will contribute to global food security and sustainable development, fostering more resilient food systems worldwide.

## Figures and Tables

**Table 1:** Smart packaging for fruit and vegetables (with respect and courtesy of the authors of all cited articles for the Figures).

Type	Material	Response Factor	Result	Reference
Temperature and humidity smart label	Metal ion	Humidity, temperature	Color variation: Light green-blue; green-red: 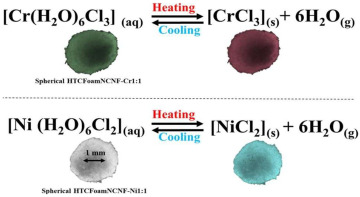	[70]
	Cresol red/thymol blue	Humidity	Color variation: lilac-yellow: 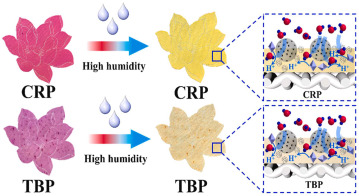	[71]
	PDA, nanoparticles	Temperature	Fruits and vegetables: AgNPs produce color changes with temperature changes to monitor fruit and vegetable quality. color variation: purple–red. 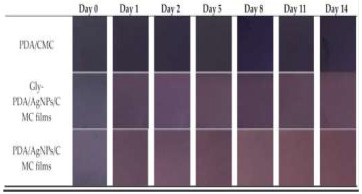	[72]
**Type**	**Smart material**	**Response factor**	**Result**	**Reference**
Integrated smart label	Visible near-infrared spectroscopy (Vis-NIR), hyperspectral imaging technique	Fruit and vegetable appearance	Apple: The method of variable iterative space contraction combined with stepwise regression was used to manage the origin and quality of apples. 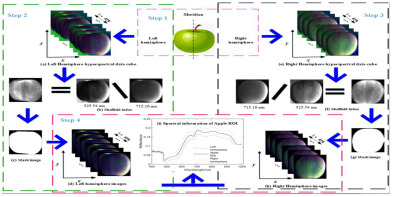	[73]
	RFID optical sensor	Color	Atlas acquisition: 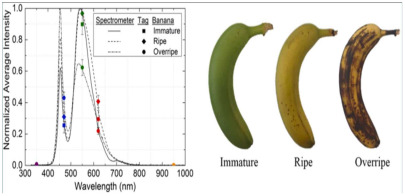	[72]
	No RFID traceability system	Position tracking	Fruit and vegetable: Use a combination of innovative RF technologies, such as RFID and NFC, and key international standards, such as EPC global, to track information about products purchased by consumers 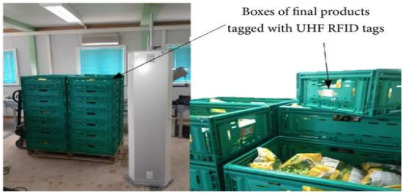	[74]
**Type**	**Smart material**	**Response factor**	**Result**	**Reference**
Smart indicating label	PEG-400	Steam	Sewant mushrooms: the freshness of fruits and vegetables is indicated by their sensitivity to water vaporcolor change: Completely opaque–transparent: 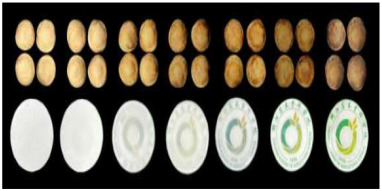	[75]
	curcumin, puerarin, and fisetin	Volatile organic compounds	Green beans, spinach, sweet corn: development and application of a 3 × 6 fluorescent sensor array that exhibits pH-sensitive properties, utilizing curcumin, puerarin, and fisetin. Volatile components are measured by this sensor. 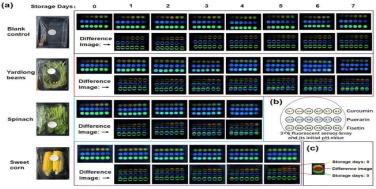	[65]
	Methyl red/bromocresol purple	Aldehyde	Kiwi fruit: developed an in-package colorimetric paper to monitor the ripeness of kiwifruit by detecting the release of aldehydes. Color changes: blue-deep red–red. 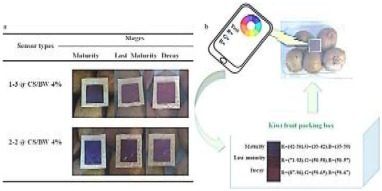	[76]
**Type**	**Smart material**	**Response factor**	**Result**	**Reference**
Active freshness-keeping packaging	Clove Essential Oil	Microorganism	Cherry tomatoes: using the antioxidant and antibacterial properties of clove essential oil, it can effectively extend the freshness life of fruits and vegetables. 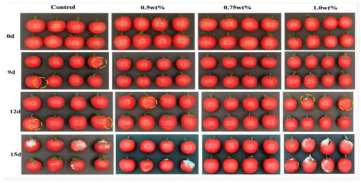	[77]
	*L. lactis*	Bacteria	Fresh-cut apples, potatoes: the incorporation of *Lactococcus lactis* (LA) significantly enhanced the antibacterial activity of the film, extending the shelf life of fruits and vegetables. 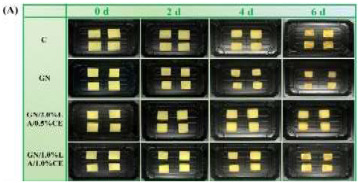	[78]
	Tree essential oil, TiO_2_	Fungus	Banana: Tea tree essential oil has anti-fungal and DPPH scavenging activity, which can effectively improve the quality of bananas. 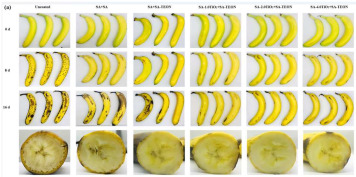	[79]

**Table 2:** Application of smart packaging in fruit and vegetable preservation (with respect and courtesy of the authors of all cited articles for the Figures).

Packaging	Purpose	Object	Reference
Ethylene-humidity regulation	Delay the softening, browning, and weight loss of mushrooms	Agaricus bisporus: 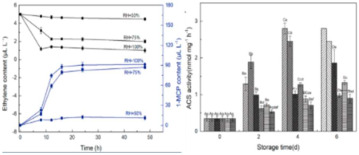	[93]
Porphyrinic metal–organic framework	High photodynamic antibacterial activity	Fresh-cut apples: 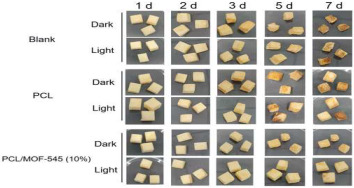	[94]
Ag-MOF nanoparticles	Preservation	Fresh apple: 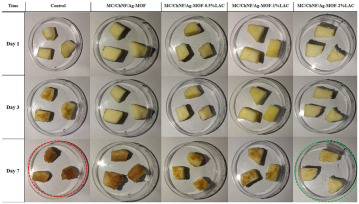	[95]
Bionic-modified atmosphere film	Preservation	Cherries and fresh-cut apple slices: 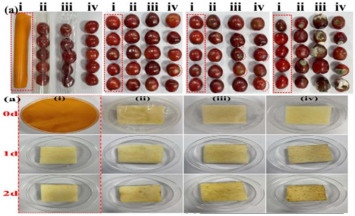	[96]
**Smart packaging**	**Purpose**	**Object**	**Reference**
Photocatalytic multifunctional film	Reduces respiration and transpiration and slows browning	Grapes and kiwifruit: 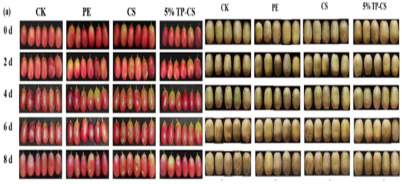	[97]
Dual-functional coatings	Anti-fog and antibacterial	Different fresh fruits and strawberries: 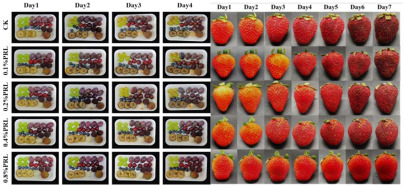	[98]
Multifunctional nanocomposite film	Antibacterial, UV resistance, and water retention properties	banana and mango: 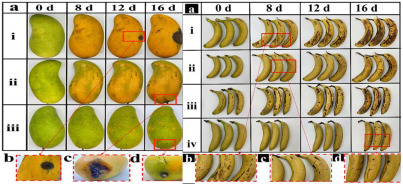	[99]

## Data Availability

No new data were created or analyzed in this study. Data sharing is not applicable to this article.

## Abbreviations

Internet of Things (IoT); time–temperature integration sensors (TTI); deep convolutional neural network (DCNN); radio frequency identification (RFID); near-field communication (NFC); electronic shelf labels (ESL); polyvinyl alcohol/carboxymethyl cellulose (PVA/CMC); chitosan (CS); sodium alginate (SA); ZIF-8 with curcumin (Cur@ZIF-8); polylactic acid (PLA); polyhydroxyfatty acid ester (PHA); montmorillonite (MMT); ε-poly(L-lysine) (ε-PL); polyvinylidene fluoride (PVDF); carbon quantum dots (CQDs); modified atmosphere packaging (MAP); 1-methylcyclopropene (1-MCP); polycaprolactone (PCL); silver metal–organic framework (Ag-MOF); lactoferrin (LAC).
